# Design and Simulation of High-Performance D-Type Dual-Mode PCF-SPR Refractive Index Sensor Coated with Au-TiO_2_ Layer

**DOI:** 10.3390/s24186118

**Published:** 2024-09-22

**Authors:** Xin Ding, Qiao Lin, Mengjie Wang, Shen Liu, Weiguan Zhang, Nan Chen, Yiping Wang

**Affiliations:** 1 Key Laboratory of Optoelectronic Devices and Systems of Ministry of Education/Guangdong Province, College of Physics and Optoelectronic Engineering, Shenzhen University, Shenzhen 518060, China; danielxin2019@126.com (X.D.); qiaolin3328@163.com (Q.L.); shenliu@szu.edu.cn (S.L.); 2 Guangdong Laboratory of Artificial Intelligence and Digital Economy (SZ), Shenzhen 518107, China; ypwang@szu.edu.cn; 3 Guangdong and Hong Kong Joint Research Centre for Optical Fiber Sensors, Shenzhen University, Shenzhen 518060, China; 4 School of Optical-Electrical and Computer Engineering, University of Shanghai for Science and Technology, Shanghai 200093, China; 221240076@st.usst.edu.cn; 5 School of Electrical Engineering and Automation, Nantong University, Nantong 226019, China; ntu_chennan@ntu.edu.cn

**Keywords:** D-type photonic crystal fiber, fundamental mode and second-order mode, surface plasmon resonance, refractive index sensor, mid-infrared waveband

## Abstract

A novel surface plasmon resonance (SPR) refractive index (RI) sensor based on the D-type dual-mode photonic crystal fiber (PCF) is proposed. The sensor employs a side-polished few-mode PCF that facilitates the transmission of the fundamental and second-order modes, with an integrated microfluidic channel positioned directly above the fiber core. This design minimizes the distance to the analyte and maximizes the interaction between the optical field and the analyte, thereby enhancing the SPR effect and resonance loss for improved sensing performance. Au-TiO_2_ dual-layer material was coated on the surface of a microfluidic channel to enhance the penetration depth of the core evanescent field and tune the resonance wavelength to the near-infrared band, meeting the special needs of chemical and biomedical detection fields. The finite element method was utilized to systematically investigate the coupling characteristics between various modes and surface plasmon polariton (SPP) modes, as well as the impact of structural parameters on the sensor performance. The results indicate that the LP_11b_y_ mode exhibits greater wavelength sensitivity than the HE_11_y_ mode, with a maximum sensitivity of 33,000 nm/RIU and an average sensitivity of 8272.7 nm/RIU in the RI sensing range of 1.25–1.36, which is higher than the maximum sensitivity of 16,000 nm/RIU and average sensitivity of 5666.7 nm/RIU for the HE_11b_y_ mode. It is believed that the proposed PCF-SPR sensor features both high sensitivity and high resolution, which will become a critical device for wide RI detection in mid-infrared fields.

## 1. Introduction

Surface plasmon resonance (SPR) occurs when incident light synchronizes with surface plasma waves, a condition influenced by the refractive index (RI) of the metal and adjacent materials. This phenomenon enables SPR technology to detect molecular binding to metal surfaces by monitoring RI changes [1,2], a capability leveraged across environmental monitoring [3], chemical detection [4,5,6], biomedicine [7], food safety [8,9,10], gas and liquid detection [11,12,13], medical diagnosis [14,15] and other fields. Photonic crystal fiber (PCF) has the advantages of a large mode field area, flexible control and high birefringence due to its unique microstructure. The fusion of PCF with SPR technology thus yields sensors of superior sensitivity. In 2006, Hassani et al. first proposed the PCF-SPR sensor, which is coated with a metal layer on the surface of the internal air hole to excite surface plasmon waves. The RI sensitivity of the sensor can reach 1000 nm/RIU and the resolution can reach 10^−4^ RIU [16]. Here, “RIU” stands for “Refractive Index Unit”. It is a unit used to express changes in the refractive index, which is a critical parameter in the field of optical sensors. In 2011, Guan et al. improved and redesigned a hexagonal RI sensor based on the original PCF-SPR sensor [17]. This structure only has one layer of air holes, reducing the difficulty and cost of sensor production, and its RI sensitivity reached over 1000 nm/RIU. In 2019, Li et al. developed an SPR sensor with a gold/graphene dual coating. Combining the advantages of graphene, the sensors exhibited higher sensitivity. The results indicated that the sensitivity of the gold layer was 1900 nm/RIU and the sensitivity of graphene layer was 2290 nm/RIU [18]. In 2020, Shafkat et al. designed a dual-core PCF-SPR sensor that achieved a wavelength sensitivity of 10,700 nm/RIU in the RI range of 1.39 to 1.40 [19]. In the same year, Yasli et al. proposed a dual-channel PCF-SPR sensor using gold and silver layers as plasma materials. The sensitivity of channels 1 and 2 of the sensor were 4100 nm/RIU and 3820 nm/RIU, respectively [20].

D-type fibers are prevalent in fiber SPR RI sensing due to their robust evanescent fields. They also boast straightforward fabrication, high mechanical stability and a side-polishing plane that facilitates the deposition of surface plasmon functional material films such as metallic coatings. In 2012, Ming et al. designed a D-type microstructure fiber SPR sensor [21] which excited the SPR by a silver plating on the polished surface. The sensitivity could reach 7300 nm/RIU and its RI detection range was between 1.33 and 1.38. In 2014, An et al. proposed a D-type microstructure fiber SPR sensor based on a rectangular arrangement of air holes [22]. The results showed that the RI detection range was between 1.35 and 1.41, and the sensitivity could reach 8129 nm/RIU. In 2018, a D-type PCF-SPR sensor based on a gold/graphene oxide composite film was proposed [23], with a maximum RI sensitivity of 10,693 nm/RIU. In the same year, Paul et al. proposed a dual-core PCF-SPR sensor with a maximum sensitivity of 25,000 nm/RIU when the RI of the measured liquid was 1.38 [24]. In 2019, Sakib et al. proposed a highly sensitive dual-core D-type PCF sensor [25]. By optimizing the structural parameters and gold film thickness, a sensitivity of 8000 nm/RIU was achieved. In 2020, Melwin et al. designed a D-type PCF with V-groove channels, with a maximum sensitivity of 31,600 nm/RIU in the range of 1.33 to 1.43 [26]. In 2021, Kiroriwal et al. proposed a PCF-SPR sensor consisting of 36 air holes, achieving a sensitivity of 8000 nm/RIU in the RI range of 1.36 to 1.40 [27]. Singh proposed a D-type PCF-SPR sensor with a dual-core symmetrical polished surface [28]. The results indicated that the maximum RI resolution could reach 4.37 × 10^−6^ RIU.

At present, SPR RI sensors developed based on D-type fiber mostly use single-mode fiber (SMF) and multi-mode fiber (MMF) structures. However, SMF, with its single-core mode, faces challenges in consistently exciting a stable SPR effect due to the stringent coupling requirements with the surface plasmon polariton (SPP) mode. Moreover, MMF will excite many SPP modes due to its numerous transmission modes. The varying coupling efficiencies between these modes and the SPP modes result in a significant broadening of the SPR resonance spectral lines, adversely affecting the sensor’s sensing performance. The D-type PCF-SPR RI sensor has also been deeply studied in theoretical simulations and experimental development, but the fundamental mode is generally used to explore its SPR sensing characteristics, ignoring the special sensitivity of higher-order modes to external RI changes. Compared to the fundamental mode, the second-order or higher-order mode in few-mode fiber tends to be more cladding in the mode field distribution, with a stronger evanescent field and penetration depth than the fundamental mode, making them more prone to strong interactions with external environmental media. With the advent of innovative fiber devices like mode division multiplexing and mode selectors, it is now feasible to selectively excite, manipulate and control specific high-order modes within few-mode fibers [29,30]. This development paves the way for leveraging second-order or higher-order modes in few-mode fibers for SPR sensing, offering a promising avenue for enhanced detection capabilities. Based on this, a D-type dual-mode PCF-SPR RI sensor is proposed in this paper. The RI sensing characteristics of the fundamental mode and second-order mode are studied, respectively, and the sensing performance of each order mode is compared, so as to achieve high-sensitivity dual-mode sensing in the near-infrared band with a wide RI detection range.

## 2. Theoretical Modeling

**Figure 1:**
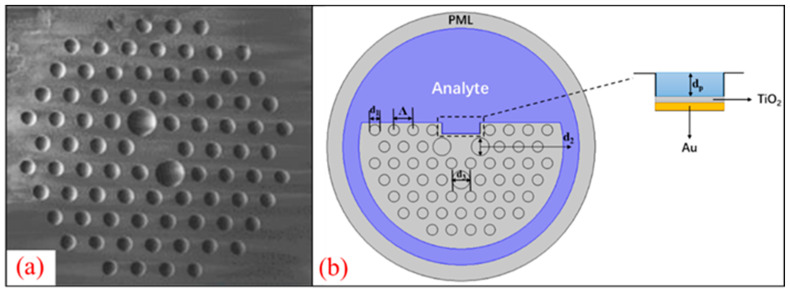
(**a**) Dual-mode PCF physical image; (**b**) Cross-section of the proposed D-type dual-mode PCF-SPR structure.

In this paper, the RI sensing characteristics of the D-type dual-mode PCF-SPR sensor are studied. In order to ensure the dual-mode characteristics of the PCF, the structure has been redesigned based on the existing dual-mode fiber. Figure 1a shows the SEM physical image of the dual-mode PCF cross-section. The cross-section of the redesigned D-type dual-mode PCF-SPR RI sensor is shown in Figure 1b. The dual-mode PCF features side-polishing and incorporates a rectangular groove near the core, serving as a microfluidic channel. The spacing between the air holes Λ is 6 μm, the diameter d_1_ of the small air hole is 3.35 μm, the diameter d_2_ of the large air hole on both sides of the core is 5.5 μm and the depth of the slot is d_p_ = 3.5 μm. In order to increase the birefringence and enhance the binding of the fiber core to the light field energy, a large air hole with a diameter of 5.5 μm (d_3_) was set directly below the fiber core. The design of the rectangular microgrooves can strengthen the penetration depth of the fiber core mode evanescent field and promote the coupling between the core mode and SPP mode. In addition, the metal film coating area and the production cost are both reduced. The metal film adopts a Au-TiO_2_ double-layer structure, and the initial thickness t_1_/t_2_ of the Au and TiO_2_ are both 50 nm.

The base material of the D-type dual-mode PCF-SPR RI sensor is fused silica, and the material dispersion is defined by the Sellmeier model [31] as follows:

(1) $$n(\lambda )=\sqrt{1+\frac{A_{1}\lambda^{2}}{\lambda^{2}-B_{1}}+\frac{A_{2}\lambda^{2}}{\lambda^{2}-B_{2}}+\frac{A_{3}\lambda^{2}}{\lambda^{2}-B_{3}}}$$

where A_1_ = 0.691663, A_2_ = 0.4079426, A_3_ = 0.8974790, B_1_ = 0.0684043 μm^2^, B_2_= 0.1162414 μm^2^, B_3_ = 9.8961610 μm^2^ and λ represents the wavelength of the incident light.

The material dispersion of the Au is described by the Drude–Lorentz model [32] as follows:

(2) $$\varepsilon \left(\omega \right)=\varepsilon_{1}+i\varepsilon_{2}=\varepsilon_{\infty }-\frac{\omega_{p}^{2}}{\omega (\omega +i\omega_{c})}$$

where $\varepsilon_{\infty }$ is the high-frequency dielectric constant of 9.75, ω is the incident optical angular frequency, $\omega_{p}$ is the gold plasma frequency of 1.36 × 10^16^ and $\omega_{c}$ is the electron scattering frequency of 1.45 × 10^14^.

TiO_2_ was used as an adhesive layer to bind the plasmonic metal to the fiber surface. TiO_2_ improves the coupling between the fundamental mode and plasmon modes, in addition to acting as an adhesive agent. TiO_2_ film can adjust the working wavelength of the SPR sensor to the near-infrared band [33]. Compared with the visible light band, the evanescent field in the near-infrared band has a higher penetration depth, which can effectively improve the sensitivity of the SPR RI sensor. The RI numerical model of TiO_2_ is as follows [34]:

(3) $$n=\sqrt{5.913+\frac{0.2441}{\lambda^{2}-0.0803}}$$

In order to evaluate the sensor performance, COMSOL Multiphysics software (version 5.6) was used for the finite element analysis. PML was used as a perfectly matched layer for the scattering and absorption. The RI of the PML was the same as the coating material, as shown in the gray part in Figure 1b. The adsorption effect of the PML mainly depends on the mesh density of the PML region. It is generally believed that a grid with five or more layers is sufficient to absorb scattered light. This model controls the maximum size of the triangular mesh within 1/5 of the wavelength range, and sets the PML to 5 μm to ensure that there are more than 5 layers of grids in the PML area, making the simulation results of the model more accurate. When light propagates axially into the redesigned dual-mode PCF, it will excite various transmission modes of different orders, and the confinement loss of each mode is expressed as follows [35]:

(4) $$\alpha_{loss}=8.686\times \frac{2\pi }{\lambda }Im\left(n_{eff}\right)\times 10^{4}\;(dB/cm)$$

where $\alpha_{loss}$ is the modal loss, $Im\left(n_{eff}\right)$ is the imaginary part of the effective RI of various transmission modes and λ indicates the wavelength of the incident light, and its unit is μm. Confinement loss refers to the lateral leakage of light during longitudinal transmission, manifested as the imaginary part of the effective RI. The confinement loss in the PCF is caused by the combination of air holes in the cladding and the internal structure of the fibers.

## 3. Model and Structural Parameter Analysis

### 3.1. Model Analysis

**Figure 2:**
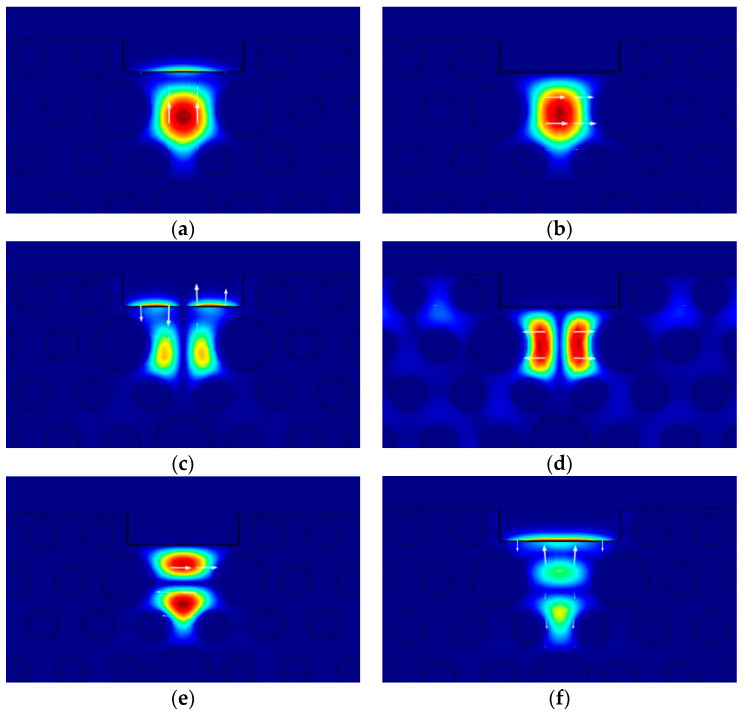
Core mode field distribution at an operating wavelength λ = 1.4 μm: (**a**) HE_11_y_ mode; (**b**) HE_11_x_ mode; (**c**) LP_11a_y_ mode; (**d**) LP_11a_x_ mode; (**e**) LP_11b_y_ mode; (**f**) LP_11b_x_ mode. (arrow indicates the electric field direction; color legend refers to the electric filed intensity).

The mode analysis of the designed D-type dual-mode PCF-SPR sensor was carried out by the finite element method. The RI of the measured analyte was set to 1.34, d_p_ = 3.5 μm. Figure 2 shows the core mode field distribution at an operating wavelength λ = 1.4 μm. The D-type dual-mode PCF supports only the LP_01_ and LP_11_ modes. The LP_01_ mode is the basic mode, which is divided into the HE_11_x_ mode and HE_11_y_ mode, as shown in Figure 2a,b. The loss of the HE_11_x_ mode is relatively small, and there is almost no mutual coupling with the SPP mode. The HE_11_y_ mode, due to its polarization direction perpendicular to the surface of the metal film, generates strong surface plasmon resonance, and part of the energy at the fiber core is transferred to the SPP mode propagating near the interface between the metal film and the measured analyte. Therefore, the loss generated by the HE_11_y_ mode is much greater than that of the HE_11_x_ mode, and only the HE_11_y_ mode in the fundamental mode can effectively excite the SPP mode and generate resonance. The second-order mode LP_11_ is divided into four modes, namely, LP_11a_x_, LP_11a_y_, LP_11b_x_ and LP_11b_y_, as shown in Figure 2c–e. Both the LP_11a_y_ and LP_11b_y_ modes are strongly coupled with the SPP mode, while second-order modes in the x direction are difficult to excite surface plasmon resonance. Therefore, it is only necessary to analyze the mode-coupling characteristics and loss spectra of the HE_11_y_, LP_11a_y_ and LP_11b_y_ modes.

**Figure 3:**
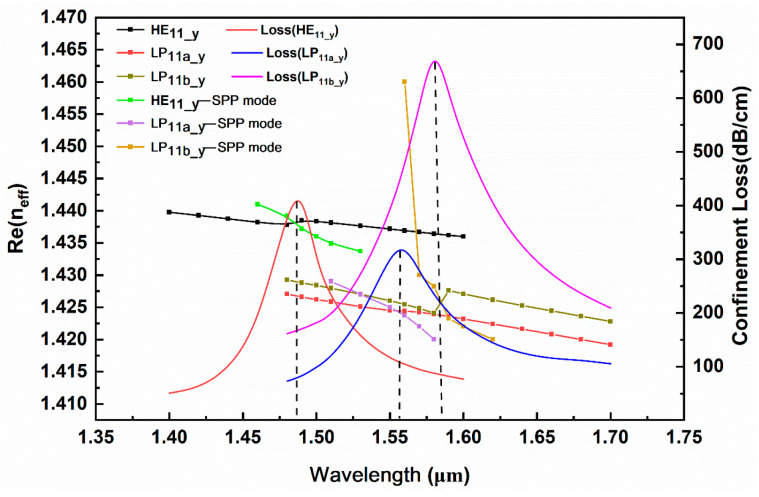
Dispersion relationship between the y polarization core modes and the corresponding SPP modes when the RI of measured analyte is 1.34.

**Figure 4:**
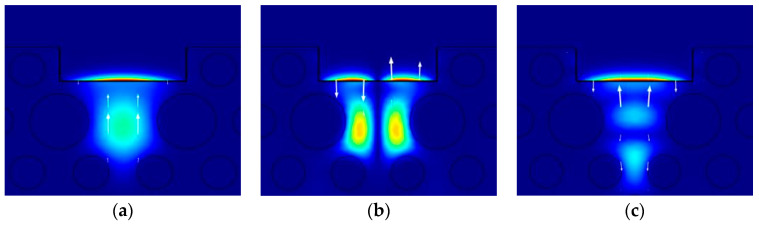
Mode field distribution when phase matching occurs between the core mode and the SPP mode: (**a**) HE_11_y_ mode; (**b**) LP_11a_y_ mode; (**c**) LP_11b_y_ mode. (arrow indicates the electric field direction; color legend refers to the electric field intensity).

Figure 3 shows the dispersion relationship curves between the core mode and the SPP mode in the y polarization direction, as well as the loss changes in each mode with the wavelength. The dot line represents the real part of the effective RI of each core mode and the corresponding SPP mode, while the solid line represents the confinement loss of the core mode. The intersection point between the real part curve of the effective RI of the core mode and the real part curve of the effective RI of the SPP mode is the phase-matching point. At this point, the effective RI of the core mode undergoes a jump, and the coupling degree between the core mode and the SPP mode is the strongest. The confinement loss of the core mode reaches its maximum, resulting in a sharp resonance peak. The real part of the fundamental mode HE_11_y_ effective RI is equal to that of the SPP mode at λ = 1.48 μm, and the resonance degree is the strongest. Figure 4a shows the mode field distribution of the fundamental mode HE_11_y_ when phase matching occurs. The resonance wavelengths of the second-order modes LP_11a_y_ and LP_11b_y_ are close to each other, and strong coupling with the SPP mode occurs at 1.56 μm and 1.58 μm, respectively. The fiber core mode field when phase matching is generated is shown in Figure 4b,c. In Figure 3, the confinement loss of the LP_11a_y_ mode is smaller than that of the LP_11b_y_ mode in the analysis band, so the loss spectrum of the LP_11a_y_ mode is actually covered by the loss spectrum of the LP_11b_y_ mode, and the SPR effect generated by the LP_11b_y_ mode is stronger. Therefore, for the second-order mode, the RI sensing characteristics of the LP_11b_y_ mode are mainly analyzed.

### 3.2. Structural Parameter Analysis

The performance of the PCF-SPR sensors is highly sensitive to changes in structural parameters. In order to further explore the influence of the designed model structural parameters in the sensor performance, this section will focus on analyzing the effects of a large air hole diameter d_3_, gold film thickness t_1_, TiO_2_ thickness t_2_ and microgroove depth d_p_ on the sensor loss spectrum when the environmental medium RI is 1.34.

(1) Large air hole diameter d_3_

**Figure 5:**
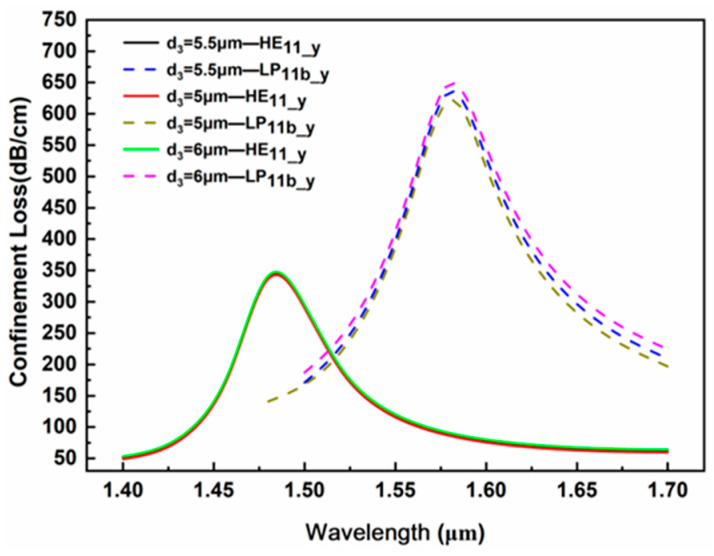
Influence of large air hole diameter d_3_ in the middle region of the PCF on the loss spectrum.

The large air hole located directly below the fiber core effectively enhances the energy confinement of the y polarization mode by the core, and its diameter affects the confinement loss of the core mode. Figure 5 shows the confinement loss corresponding to different large air hole diameters d_3_ under the conditions that the measured analyte RI is 1.34, the gold film thickness t_1_ is 50 nm, the TiO_2_ thickness t_2_ is 50 nm and microgroove depth d_p_ is 3.5 μm. For the fundamental mode HE_11_y_, the change in d_3_ hardly affects the confinement loss of the HE_11_y_ mode, and its resonance peak position also hardly shifts. Therefore, the influence of d_3_ on the fundamental mode HE_11_y_ is negligible. Compared to the fundamental mode HE_11_y_, the mode field of LP_11b1_y_ has stronger energy diffusion in the y polarization direction, and its evanescent field penetration depth is greater. With the increase in d_3_, the confinement loss of the LP_11b1_y_ mode is enhanced. This is because the increase in d_3_ enhances the energy binding of the fiber core to the LP_11b_y_ mode, allowing more energy to couple with the SPP mode, resulting in stronger surface plasmon resonance effects and increasing the confinement loss. However, overall, d_3_ has a relatively small impact on the confinement loss and resonance peak position of the LP_11b_y_ mode, and the amplitude of loss variation remains within the range of 30 dB/cm, with almost no shift in the position of the resonance peak on the whole. The diameter d_2_ of the air holes on both sides of the fiber core has been regulated at 5.5 μm. To reduce the manufacturing difficulties of the PCF and eliminate the inconsistency of the air hole diameters, this design adopts d_3_ = d_2_ = 5.5 μm as one of the optimum sensor structural characteristics.

(2) Gold film thickness t_1_

**Figure 6:**
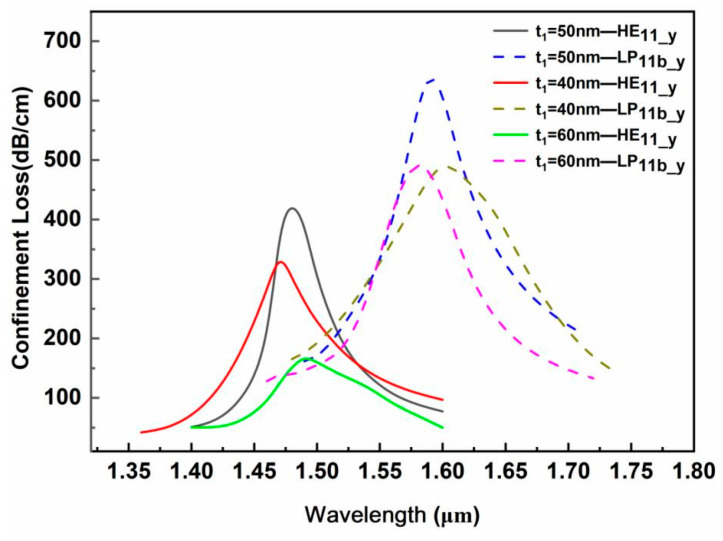
Influence of the gold film thickness t_1_ on the loss spectrum.

The variation in the gold film thickness has a significant impact on the loss spectrum of various modes. Surface plasmon waves are very sensitive to changes in the thickness of the metal layer, and the phase-matching point and coupling efficiency of the fiber core mode and the SPP mode are modulated by the thickness of the metal layer. Figure 6 shows the confinement loss corresponding to different gold film thicknesses t_1_ under the conditions that the measured analyte RI is 1.34, the large air hole d_3_ is 50 nm, the TiO_2_ thickness t_2_ is 50 nm and microgroove depth d_p_ is 3.5 μm. For the base mode HE_11_y_, as the gold film thickness increases, its resonance peak undergoes a small red shift, and its confinement loss first increases and then decreases. The fundamental mode confinement loss corresponding to a gold film thickness of 50 nm is the strongest, and its resonance loss peak broadening is the smallest. For the LP_11b_y_ mode, as the gold film thickness increases, its resonance peak undergoes a blue shift. At a gold film thickness of 50 nm, the coupling between the LP_11b_y_ mode and the SPP mode is the strongest, resulting in strong surface plasmon resonance. On the other hand, as the gold film thickness increases, the broadening of the resonance peak of the LP_11b_y_ mode gradually decreases. Considering that the resonance peak of the integrated fundamental mode HE_11_y_ has the strongest resonance and the smallest resonance peak broadening at a gold film thickness of 50 nm, a gold film thickness of t_1_ = 50 nm is selected as the optimized structural parameter.

(3) TiO_2_ thickness t_2_

**Figure 7:**
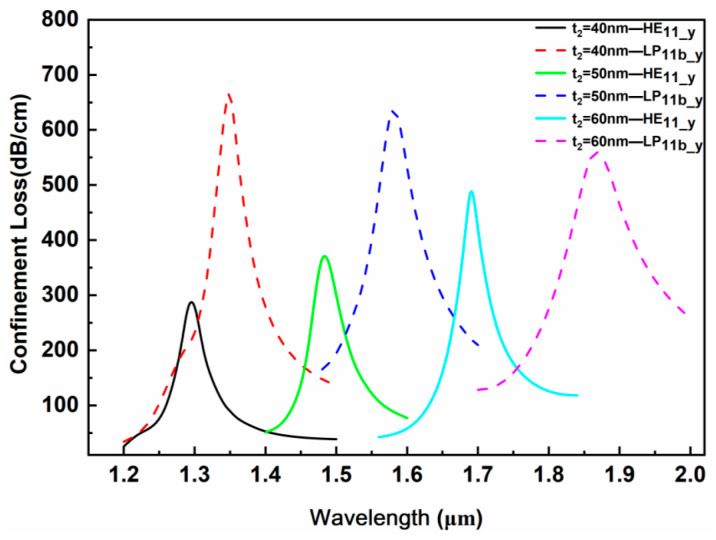
Influence of the TiO_2_ thickness t_2_ on the loss spectrum.

The size of TiO_2_ has a significant wavelength-tuning effect on the position of the SPR resonance peaks. Figure 7 shows the confinement loss corresponding to different TiO_2_ thicknesses t_2_ under the conditions that the measured analyte RI is 1.34, the large air hole d_3_ is 50 nm, the gold film thickness t_1_ is 50 nm and the microgroove depth d_p_ is 3.5 μm. The resonance peak wavelength of the HE_11_y_ mode has a red shift with the increase in the TiO_2_ thickness. For every 10 nm increase in thickness, the resonance peak position shifts towards the longer wavelength direction by nearly 200 nm. The corresponding loss peak intensity of the HE_11_y_ mode increases, and the broadening of the resonance peak varies little. For the second-order mode LP_11b_y_, the resonance peak wavelength also has a red shift with the increase in the TiO_2_ thickness, but the intensity of the resonance peak gradually decreases and the broadening of the resonance peak increases. When the thickness of TiO_2_ is 40 nm, the loss spectrum of the LP_11b_y_ mode is highly overlapped with that of the HE_11_y_ mode, and the characteristic peaks corresponding to the HE_11_y_ mode are covered by the LP_11b_y_ mode, which makes the dual-mode sensing characteristics of the designed sensor unclear. Therefore, t_2_ = 40 nm is not the optimal structural parameter. Due to the superior resonance peak intensity and broadening of the LP11b1_y mode loss spectrum, corresponding to t_2_ = 50 nm, compared to the LP11b1_y mode loss spectrum, corresponding to t_2_ = 60 nm, a TiO_2_ thickness of t_2_ = 50 nm was comprehensively considered as the optimized sensor structural parameter.

(4) Microgroove depth d_p_

**Figure 8:**
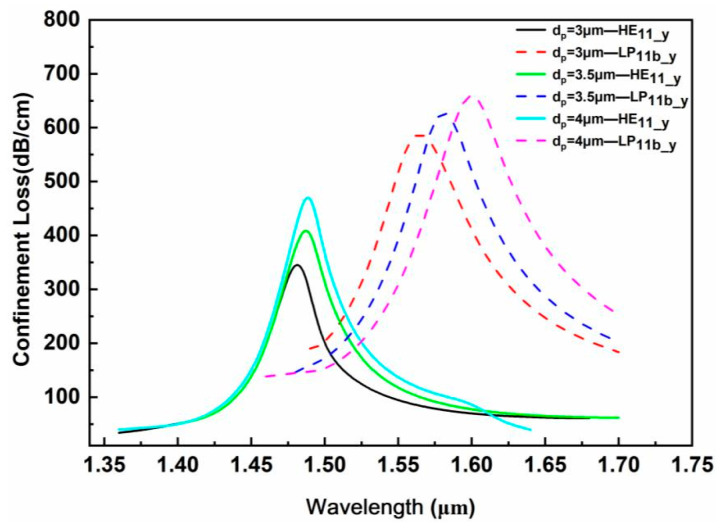
Influence of the microgroove depth d_p_ on the loss spectrum.

Figure 8 shows the confinement loss corresponding to different microgroove depths d_p_ under the conditions that the measured analyte RI is 1.34, the large air hole d_3_ is 50 nm, the gold film thickness t_1_ is 50 nm and the TiO_2_ thickness t_2_ is 50 nm. When d_p_ increases, the resonance loss corresponding to the fundamental mode HE_11_y_ and the second-order mode LP_11b_y_ increases, and the resonance peak wavelength has a red shift. The increase in the polishing depth brings the distance between the metal layer and the fiber core closer, enhances the interaction between light and matter and makes it easier for the evanescent field to reach the interface between the metal and the measured analyte. Meanwhile, the fiber core mode and the SPP mode generate stronger mutual coupling. Although the SPR effect of each order mode is the strongest when microgroove depth is 4 μm, an excessive polishing depth will affect the mechanical stability of the fiber itself and increase the difficulty of fabrication, and, in addition, will compress the mode field area of the core mode. Therefore, a compromise is considered to choose d_p_ = 3.5 μm as one of the optimal structural parameters, where the change in d_p_ has little effect on the broadening of the resonance peak.

## 4. Analysis of RI Sensing Characteristics

The RI sensitivity of the D-type dual-mode PCF-SPR sensor proposed in this paper can be expressed by the wavelength sensitivity. Wavelength sensitivity is defined as the shift of the resonance wavelength relative to the RI change. The wavelength sensitivity formula is as follows [36]:

(5) $$S_{\lambda }=\Delta_{res}/{\Delta n}_{\alpha }$$

where $\Delta_{res}$ represents the shift of the resonance wavelength and ${\Delta n}_{\alpha }$ represents the change in the measured analyte RI.

The sensor resolution formula is as follows [37]:

(6) $$R\left(RIU\right)={\Delta n}_{\alpha }\times \frac{{∆\lambda }_{min}}{{\Delta \lambda }_{peak}}(RIU)$$

where ${∆\lambda }_{min}$ represents the minimum spectral resolution, whose value is 0.02 nm, and ${\Delta \lambda }_{peak}$ represents the offset of the resonance peak.

The Figure of Merit (*FOM*) is another important parameter for measuring the sensing performance, and its calculation formula is as follows [38]:

(7) $$FOM=\frac{S(nm\times {RIU}^{-1})}{FWHM(nm)}$$

**Figure 9:**
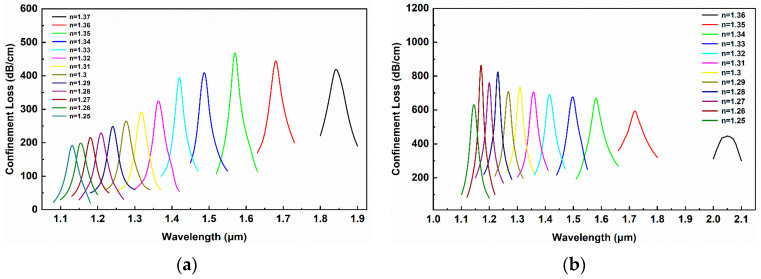
Response to RI under optimized parameters: (**a**) HE_11_y_ mode; (**b**) LP_11a_y_ mode.

Through the analysis of the influence of the structural parameters on the sensor loss spectrum in the previous section, the optimal and reasonable parameter that can obtain the optimal mode loss spectrum characteristics and ensure that the dual-mode loss peak does not overlap are d_3_ = 5.5 μm, t_1_ = 50 nm, t_2_ = 50 nm and d_p_ = 3.5 μm. Figure 9a,b show the loss spectrum curves corresponding to different RIs of the measured analyte under the above conditions. The RI detection range of the fundamental mode HE_11_y_ is 1.25 to 1.37, and the shift range of the resonance peak is 1.1 μm to 1.9 μm. As the measured analyte RI increases, a red shift occurs. When the RI changes from 1.36 to 1.37, the wavelength shift of the resonance peak reaches a maximum of 160 nm. Therefore, the maximum RI sensitivity of the fundamental mode HE_11_y_ is 16,000 nm/RIU, and the minimum RI spatial resolution can reach 6.25 × 10^−6^ RIU. For the second-order mode LP_11b_y_, its RI detection range is 1.25 to 1.36, and the shift range of the resonance peak is 1.1 μm to 2.1 μm. Similar to the fundamental mode, the resonance peak of the LP_11b_y_ mode also shifts towards the longer wavelength direction as the measured analyte RI increases. The loss peak intensity of the second-order mode is generally higher than that of the fundamental mode. When the external RI changes, the LP_11b1_y_ mode will excite stronger surface plasmon resonance, resulting in a more pronounced RI sensing response. When the RI changes from 1.35 to 1.36, the maximum wavelength shift of the loss peak corresponding to the LP_11b_y_ mode is 330 nm, which is greater than the offset of 110 nm, corresponding to the base mode HE_11_y_. Therefore, the maximum RI sensitivity of the LP_11b_y_ mode is 33,000 nm/RIU, and the minimum RI spatial resolution is 3.03 × 10^−6^ RIU.

**Figure 10:**
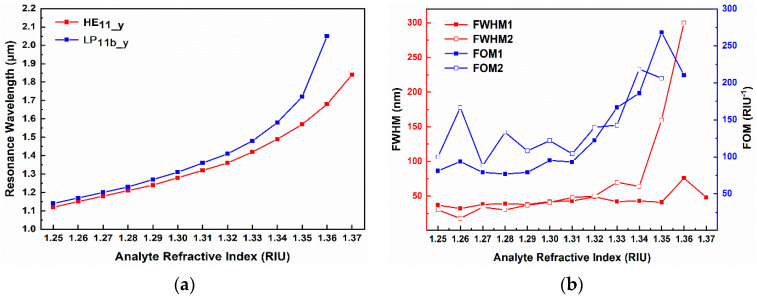
(**a**) Relation between the RI and resonance wavelength; (**b**) *FWHM* and *FOM*.

The relationship between the resonant wavelength and the RI of the D-type dual-mode PCF-SPR RI sensor is shown in Figure 10a, and the slope represents the RI sensitivity of each mode. In the RI range of 1.25 to 1.32, the resonant wavelengths of the fundamental mode HE_11_y_ and the second-order mode LP_11b_y_ both exhibit a good linear growth relationship with the RI. The slope of the LP_11b_y_ curve is slightly higher than that of the fundamental mode HE_11_y_, indicating that the RI sensitivity of the LP_11b_y_ mode is higher than the fundamental mode HE_11_y_. Within the RI range of 1.33 to 1.37, the RI sensitivity of each dual-mode gradually increases, and the further the distance between the resonant peak wavelengths, the more two resonant peaks will appear in the loss spectrum, which is conducive to achieving the dual-peak resonant sensing of the HE_11_y_ and LP_11b_y_ modes. Through calculation, the average RI sensitivity of the HE_11_y_ mode is 5666.7 nm/RIU over the whole range of RI variation. The RI detection ranges of the LP_11b_y_ mode is from 1.25 to 1.36, with an average RI sensitivity of 8272.7 nm/RIU, which is much higher than the RI sensitivity of the fundamental mode. Figure 10b shows the relationship between the full width at half maximum (*FWHM*) and the *FOM* of the resonance peaks corresponding to two modes as a function of the RI.

When the RI is in the range of 1.25 to 1.33, the half-width *FWHM2* corresponding to the LP_11b_y_ mode and the half-width *FWHM1* corresponding to the HE_11_y_ mode are very similar, and both are less than 50 nm, while the *FOM2* corresponding to the LP_11b_y_ mode is basically higher than the *FOM1* corresponding to the HE_11_y_ mode, and both reach high levels of 100 RIU^−1^ or above. This makes the sensing performance of the LP_11b_y_ mode better than that of the base mode HE_11_y_ and has a better signal-to-noise ratio. When the RI exceeds 1.34, the *FWHM* of the LP_11b_y_ mode resonance peak significantly increases, especially when the RI reaches 1.36 and the broadening reaches over 300 nm. At this time, the RI sensing performance deteriorates. Therefore, the LP_11b_y_ mode is no longer suitable for the sensing and detection of the RI exceeding 1.36. The resonance peak broadening of the fundamental mode in the RI range of 1.34–1.37 is much smaller than that of the LP_11b_y_ mode, and the *FOM* is higher, with the minimum being greater than 186.05 RIU^−1^.

Table 1 compares the main sensing performance parameters of some existing D-type PCF-SPR RI sensors with the sensors proposed in this paper. By comparison, the D-type dual-mode PCF-SPR sensor designed in this paper has a very wide range of RI sensing and detection capabilities, especially for detecting a low RI below 1.33. In addition, the wavelength detection range covers the near-infrared band of 1100–2000 nm, which can fully meet the needs of large-scale RI sensing in the near-infrared band. Wavelength sensitivity is one of the most important sensing properties. The proposed D-type dual-mode sensor has a relatively higher wavelength sensitivity in both the fundamental and second-order modes, with the maximum sensitivity of the second-order mode reaching over 30,000 nm/RIU. The smaller detection resolution and higher FOM are also advantages of the sensor designed in this paper.

## 5. Conclusions

In this paper, an SPR RI sensor based on the D-type dual-mode PCF is presented and simulated by the finite element method. On the basis of the D-type PCF, a microgroove is designed above the fiber core as a microfluidic channel, which not only makes the tested material medium closer to the core to produce a stronger SPR effect, but also reduces the amount of measured analyte and the area of the metal coating. Gold film is used as the material to excite the SPR effect, and a TiO_2_ film is added on top of the gold film to adjust the working wavelength to the near-infrared band to meet the needs of chemical and biomedical detection. The results show that both the fundamental mode and the second-order mode in the y polarization direction can effectively excite the SPR effect, while the coupling between the core mode and the SPP mode in the x polarization direction is very weak. The resonance wavelengths of the HE_11_y_ mode and the LP_11b_y_ mode are not consistent, and two resonance peaks will appear in the loss spectrum. Moreover, the LP_11b_y_ mode can excite stronger SPR effects, resulting in higher resonance losses. The sensing performance is affected by the diameter d_3_ of the large air hole directly below the fiber core, the depth d_p_ of the microgroove, the thickness t_1_ of the gold film and the thickness t_2_ of the TiO_2_. The optimized structural parameters are d_3_ = 5.5 μm, t_1_ = 50 nm, t_2_ = 50 nm and d_p_ = 3.5 μm. Compared with the HE_11_y_ mode and the LP_11b_y_ mode, the LP_11b_y_ mode exhibits higher wavelength sensitivity, with a maximum sensitivity of 33,000 nm/RIU and an average sensitivity of 8272.7 nm/RIU in the RI sensing range of 1.25–1.36, which is higher than the maximum sensitivity of 16,000 nm/RIU and the average sensitivity of 5666.7 nm/RIU for the the HE_11_y_ mode. The LP_11b_y_ mode has a smaller minimum detection resolution than the fundamental mode, reaching 3.03 × 10^−6^ RIU. In addition, the FOM of both modes reaches over 200, indicating that the sensor has excellent dual-mode RI sensing characteristics. This characteristic, combined with mode division multiplexing or mode-selection technology, can achieve multi-channel sensing with a high sensitivity and wide RI detection range in the near-infrared band.

## Figures and Tables

**Table 1:** Performance comparison between the proposed sensor and other D-type PCF-SPR sensors.

Refs.		RI Range	Wavelength Range (nm)	Max Sensitivity (nm/RIU)	Resolution (RIU)	FOM
[39]		1.33–1.38	2030–2310	10,493	9.53 × 10^−6^	N/A
[40]		1.33–1.37	480–650	3700	2.7 × 10^−5^	N/A
[41]		1.33–1.35	1870–2300	17,000	5.8 × 10^−6^	N/A
[42]		1.33–1.39	1400–2200	21,100	4.739 × 10^−6^	106.81
[43]		1.36–1.41	650–1440	14,660	6.82 × 10^−6^	250
[44]		1.27–1.36	550–1100	2350	2.8 × 10^−5^	N/A
[45]	x-polarized y-polarized	1.29–1.36	1150–2200 1160–2200	4157 3704	2.41 × 10^−8^	N/A
[46]		1.22–1.33	1200–2250	15,000	6.67 × 10^−6^	N/A
This work	HE_11_y_ LP_11b_y_	1.25–1.37 1.25–1.36	1120–1840 1150–2050	16,000 33,000	6.25 × 10^−6^ 3.03 × 10^−6^	268.29 206.25

## Data Availability

The data are not publicly available due to the Confidentiality and Non-disclosure Agreement with the funders.
